# Supplementary material for: Hybrid Models and Biological Model Reduction with PyDSTool
Source: PLoS Comput Biol. 2012 Aug 9;8(8):e1002628. doi: 10.1371/journal.pcbi.1002628 (PMC3415397; doi:10.1371/journal.pcbi.1002628)
Supplement: Text S4 — Complete source code for the PyDSTool package (version 0.88.120504). Includes API documentation and help files linking to web pages. This file is identical to the current public release on Sourceforge.net. (ZIP) [file pcbi.1002628.s004.zip › PyDSTool/html/PyDSTool.ModelSpec'.Component-class.html]

xml version="1.0" encoding="ascii"?


PyDSTool.ModelSpec'.Component


| Home | Trees | Indices | Help | PyDSTool |
| --- | --- | --- | --- | --- |

Package PyDSTool ::
Module ModelSpec' ::
Class Component

[hide private]

[frames] | no frames]

# Class Component

source code

```
object --+    
         |    
 ModelSpec --+
             |
            Component
```

Known Subclasses:
:   - Toolbox.neuralcomp.compatODEComponent

---

Non-leaf node sub-class of ModelSpec abstract class.


|  |  |
| --- | --- |
| Instance Methods | [hide private] |

|  |  |
| --- | --- |
| compileFuncSpec(self, ignoreInputs=False) | source code |

|  |  |
| --- | --- |
| isDefined(self, verbose=False, ignoreInputs=False) | source code |

|  |  |
| --- | --- |
| isEmpty(self) | source code |

**Inherited from `ModelSpec`**:
`__call__`,
`__contains__`,
`__copy__`,
`__deepcopy__`,
`__delitem__`,
`__eq__`,
`__getitem__`,
`__init__`,
`__ne__`,
`__repr__`,
`__str__`,
`add`,
`addConnxnTarget`,
`delConnxnTarget`,
`difference`,
`flattenSpec`,
`info`,
`isComplete`,
`remove`,
`rename`,
`search`,
`validate`

**Inherited from `ModelSpec`** (private):
`_infostr`,
`_register`

**Inherited from `object`**:
`__delattr__`,
`__getattribute__`,
`__hash__`,
`__new__`,
`__reduce__`,
`__reduce_ex__`,
`__setattr__`


|  |  |
| --- | --- |
| Class Variables | [hide private] |

compatibleContainers = `(<class 'PyDSTool.ModelSpec.Component'>)`

compatibleSubcomponents = `(<class 'PyDSTool.Symbolic.Par'>, <c...`

**Inherited from `ModelSpec`**:
`compatibleGens`,
`targetLangs`


|  |  |
| --- | --- |
| Properties | [hide private] |

**Inherited from `object`**:
`__class__`


|  |  |
| --- | --- |
| Method Details | [hide private] |

|  |  |
| --- | --- |
| compileFuncSpec(self, ignoreInputs=False) | source code |

Overrides: ModelSpec.compileFuncSpec

|  |  |
| --- | --- |
| isDefined(self, verbose=False, ignoreInputs=False) | source code |

Overrides: ModelSpec.isDefined

|  |  |
| --- | --- |
| isEmpty(self) | source code |

Overrides: ModelSpec.isEmpty

  


|  |  |
| --- | --- |
| Class Variable Details | [hide private] |

### compatibleSubcomponents

Value:
:   ```
    (<class 'PyDSTool.Symbolic.Par'>,
     <class 'PyDSTool.Symbolic.Var'>,
     <class 'PyDSTool.Symbolic.Input'>,
     <class 'PyDSTool.Symbolic.Fun'>,
     <class 'PyDSTool.ModelSpec.Component'>,
     <class 'PyDSTool.ModelSpec.LeafComponent'>)
    ```

  


| Home | Trees | Indices | Help | PyDSTool |
| --- | --- | --- | --- | --- |

|  |  |
| --- | --- |
| Generated by Epydoc 3.0.1 on Fri May 4 15:24:07 2012 | http://epydoc.sourceforge.net |
